# Breast Cancer Metastasis Masquerading as a Primary Gynecological / Colonic Malignancy: A Rare Diagnostic Conundrum

**DOI:** 10.7759/cureus.7806

**Published:** 2020-04-24

**Authors:** Humaira Sarfraz, Diana Chen, Ibrahim N Muhsen, Mary R Schwartz, Martina Ogbonna

**Affiliations:** 1 Internal Medicine, Houston Methodist Hospital, Houston, USA; 2 Pathology, Houston Methodist Hospital, Houston, USA

**Keywords:** invasive lobular breast cancer, metastatic, colorectal cancer

## Abstract

Breast cancer is the most common malignancy affecting women. Metastatic involvement of the gastrointestinal (GI) tract secondary to a primary breast malignancy is rare. Here, we describe the case of a 60-year-old woman with a history of right lobular breast cancer (diagnosed and treated five years prior to presentation) who presented with fatigue, generalized abdominal pain, distension, weight loss, and vomiting. Her initial imaging was suspicious for a primary gynecological malignancy; however, subsequent workup showed a colonic mass. Total colonoscopy revealed colon metastases, and an immunohistochemical profile favored invasive lobular carcinoma of breast. Most cases of gastrointestinal metastases from breast cancer have lobular histology; however, colonic involvement is rare.

## Introduction

Breast cancer is the most common primary malignancy second only to non-melanoma skin cancer [1-2]. Invasive lobular carcinoma accounts for only 5% to 15% of the total breast malignancies, but this still remains the most common histopathological diagnosis in the case of metastasis to the gastrointestinal (GI) tract [3-4]. While metastasis from breast cancer involves multiple body systems namely lung, liver, brain, and bone, dissemination to the GI tract is rare. A retrospective study by McLemore *et al.* showed that only 73 of the 12,001 cases had breast metastasis [5]. However, post-mortem examination shows that almost 16% of patients with breast cancer have GI metastasis. Benign disease processes or second primaries affecting the GI tract are more common than isolated gastrointestinal metastasis [6-7] This case describes a 60-year-old female with metastatic invasive lobular cancer to the colon presenting with non-specific abdominal symptoms behaving like a primary uterine malignancy on initial imaging. However, pathology revealed a primary breast origin.

## Case presentation

A 60-year-old female with a history of right lobular breast cancer presented with generalized abdominal pain and distension. Other associated features included unintentional weight loss of almost 20 lbs in the last few months, fatigue, five weeks of nausea, vomiting, poor oral intake, and odynophagia. She denied melena, hematochezia, diarrhea, or pelvic bleeding/ discharge. The last bowel movement was a day prior to her admission.

Her past medical history was significant for estrogen receptor (ER)-positive, progesterone receptor (PR)-positive, human epidermal factor 2 (HER2)-negative right breast lobular cancer diagnosed five years prior to presentation. For this, she underwent bilateral mastectomy the same year. The left breast was removed pre-emptively. The patient did not receive any chemotherapy or radiation treatment. She was started on Tamoxifen which she took for five years. She denied any tobacco use, alcohol or recreational drug use. Family history was significant for breast cancer in her sister. Other medical conditions included diabetes mellitus, hypothyroidism, and hypertension. She had never had colonoscopy prior to this admission.

Physical exam revealed stable vital signs, distended abdomen, generalized tenderness with positive fluid thrill and decreased bowel sounds.

She presented initially to an outside hospital where a CT scan of the abdomen and pelvis was performed which showed a moderate-sized right-sided pleural effusion, large ascites in the abdomen, an enlarged uterus with multiple fibroids with endometrial enhancement but no evidence of colonic thickening/ obstruction noted. She underwent thoracentesis which was consistent with an exudate (light's criteria positive) with 186 nucleated cells (48% lymphocytes and 49% macrophages). Also, the patient underwent paracentesis which showed a hazy yellow fluid with 243 nucleated cells (11% neutrophils, 4% mesothelial cells, 37% lymphocytes and 52% macrophages), Serum Albumen Ascites Gradient (SAAG) of 0.4 g/dL suspicious for malignancy versus tuberculosis (TB). Both the initial cytologies for malignancy and TB cultures were negative. Elevated tumor markers including Carcinoembryonic Antigen (CEA) (19.4), Carbohydrate Antigen (CA) 27-29 (101), CA15-3 (72) and CA125 (224) were noted. Her Complete Blood Count was remarkable for normocytic anemia with Hb 8.9 with iron studies consistent with anemia of chronic disease (elevated ferritin, decreased total iron binding capacity and reduced transferrin).

Meanwhile, she was found to be having a UTI with E. coli and a right lower extremity deep venous thrombosis for which she was started on antibiotics and heparin drip for treatment. She underwent upper endoscopy as part of her odynophagia work up which showed candida esophagitis that was treated with fluconazole.

She was also evaluated by the gynecological oncology team due to the concern for a primary uterine malignancy with likely malignant ascites. She underwent repeat paracentesis, the cytology of which was negative for malignancy.

Subsequently, MRI abdomen/ pelvis was done which revealed distal descending colon narrowing concerning for a colonic mass along with peritoneal carcinomatosis. However, enlarged multi-fibroid uterus was noted again. On the same day, the patient underwent a repeat paracentesis for symptom alleviation. The cytology results showed that the malignant cells were positive for MOC-31, BerEP4, GATA-3 (diffuse), mammaglobin (diffuse) and ER, supporting breast origin. PAX-8 was negative, pointing against a gynecologic origin. Also D2-40 and calretinin highlight background mesothelial cells. These results supported the diagnosis of metastatic carcinoma of breast origin and the morphology favoring lobular carcinoma.

**Figure 1:**
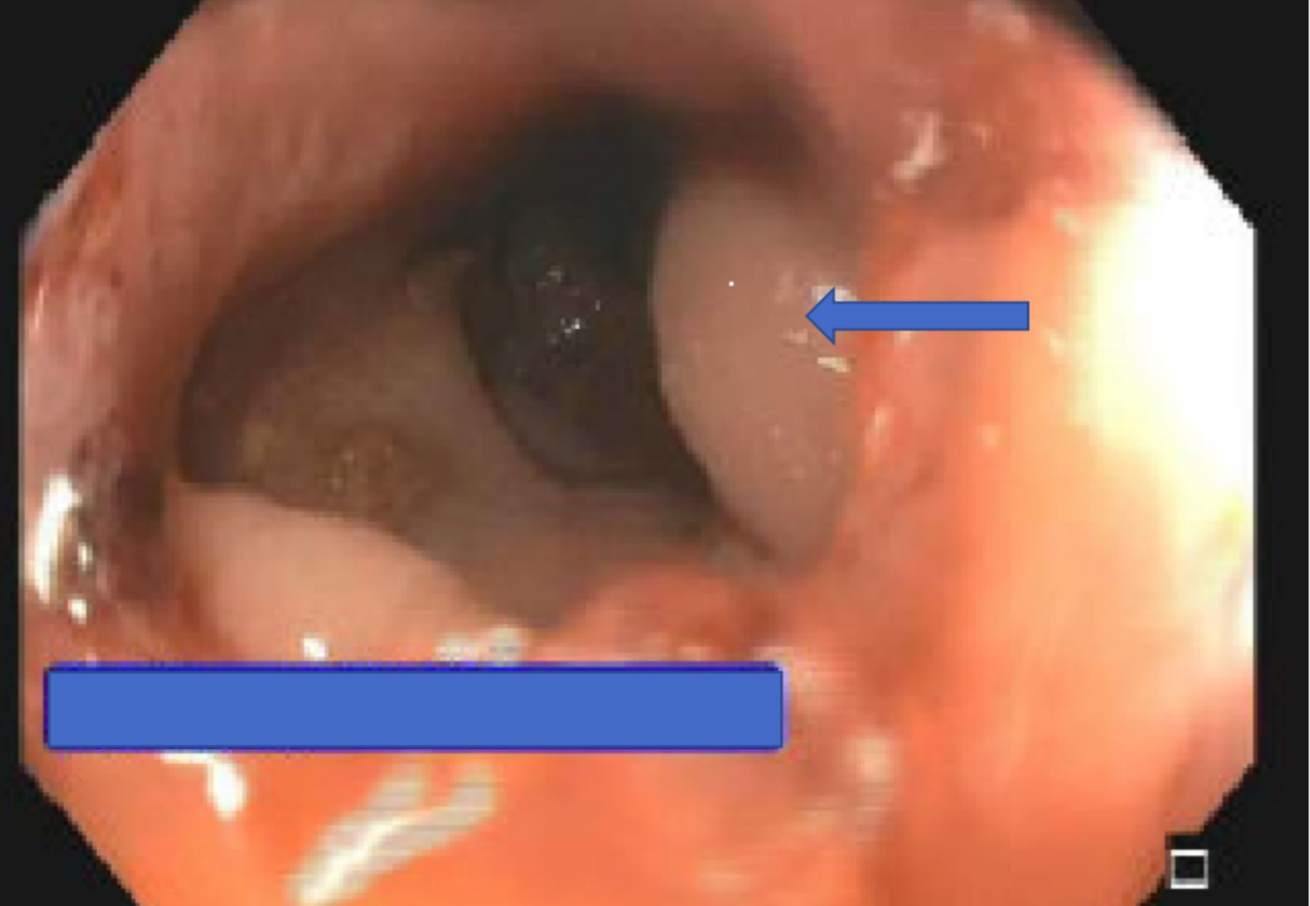
Flexible sigmoidoscopy which showed an infiltrating, circumferential partially obstructing mass in the rectosigmoid colon The mass was one cm in length with a diameter of fifteen millimeters. No bleeding was noted.

**Figure 2:**
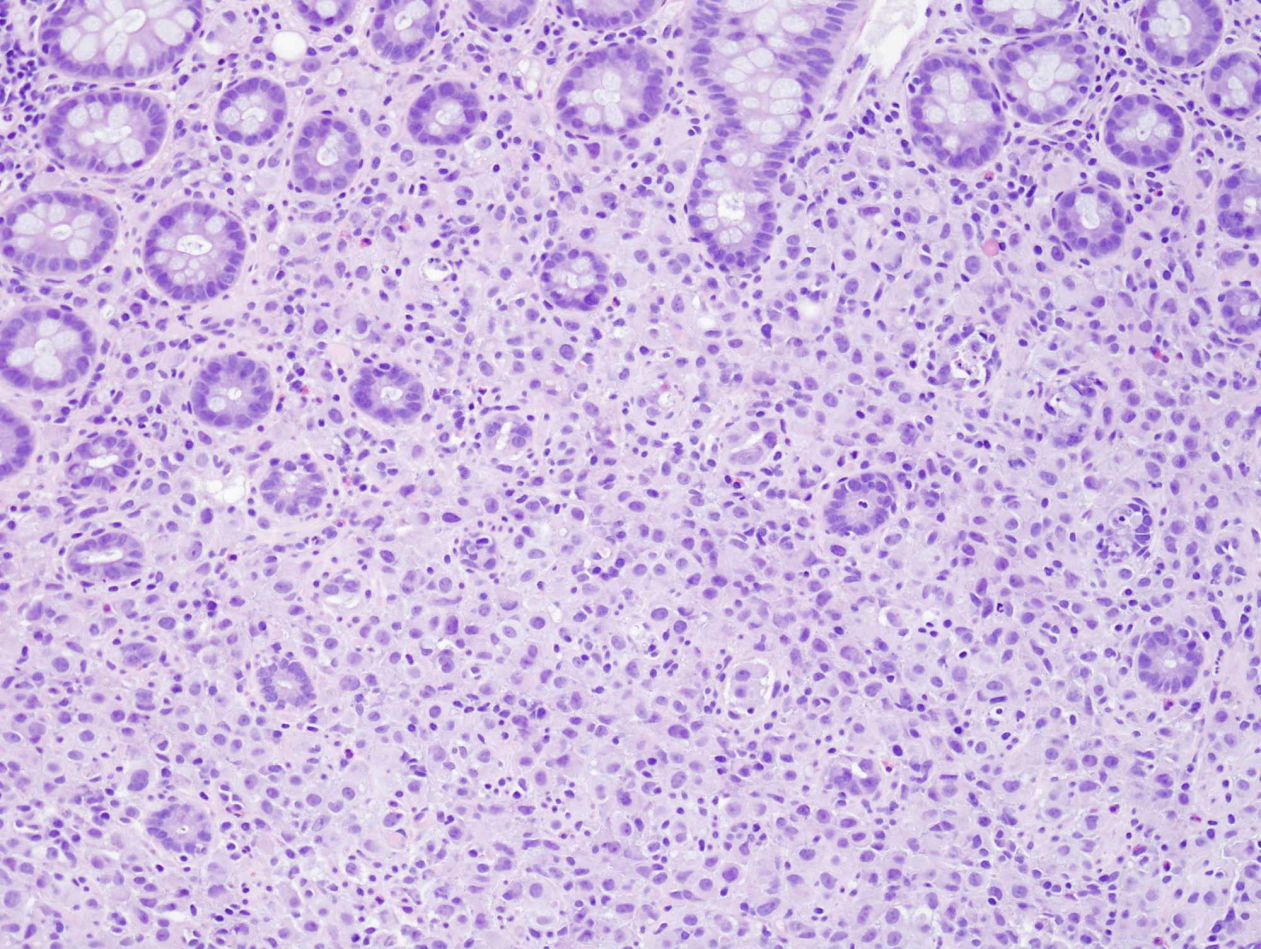
Photomicrograph of a sigmoid colon biopsy showing diffuse infiltration by neoplastic cells

**Figure 3:**
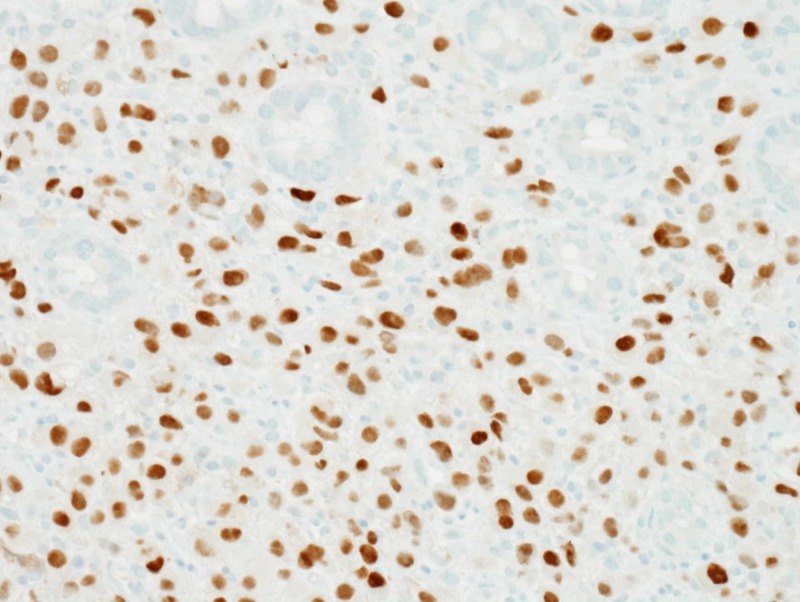
Immunohistochemical staining for ER demonstrating strong ER positivity in the tumor with the majority of tumor nuclei having positive immunostaining for ER ER, estrogen receptor

**Figure 4:**
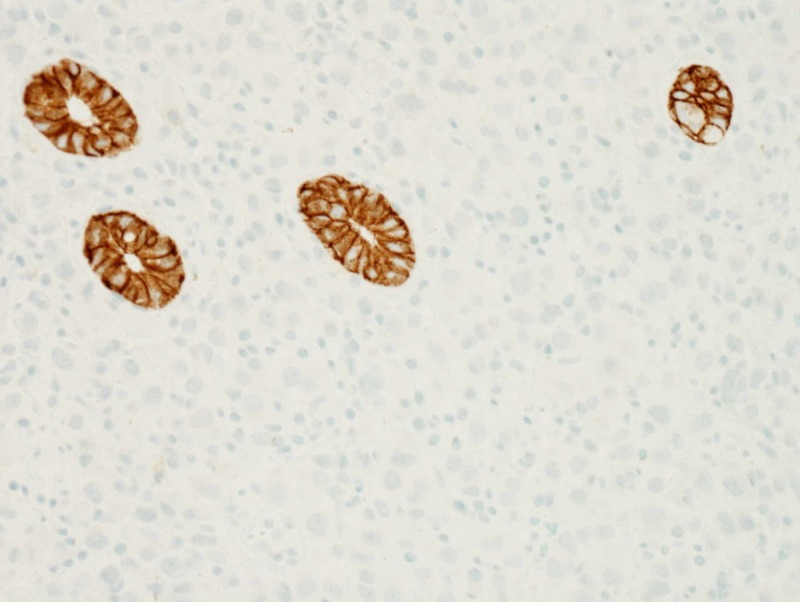
Immunohistochemical stain for E-cadherin demonstrating loss of E-cadherin expression in the carcinoma, supportive of lobular differentiation Note the preservation of E-cadherin expression in the non-neoplastic colonic crypts.

Thereafter, the patient underwent flexible sigmoidoscopy which showed an infiltrating, circumferential partially obstructing mass in the rectosigmoid colon (Figure 1). The mass was 1 cm in length with a diameter of 15 mm. No bleeding was noted. Biopsy results were consistent with invasive lobular cancer with primary breast cancer, ER-positive-5/5 (85% tumor cells), and PR-4/5 (65% tumor cells); HER2 gene amplification was negative by FISH (Figures 2, 3 and 4).

The teams discussed with the family the non-curable nature of the disease and provided information on treatment options that could achieve some measure of disease control. The patient was subsequently started on letrozole and abemciclib and discharged to a skilled nursing facility with scheduled frequent paracentesis

## Discussion

Almost one in eight US women develop breast cancer over the course of their lifetime. It is speculated by the American Cancer Society that in 2019, 1,762,450 new cancer cases and 606,880 cancer-related deaths will occur in the United States [1]. While at times breast cancer is metastatic even at initial diagnosis, it is important to consider it as a differential even if diagnosed and treated at an earlier stage. This is because the disease may recur and metastasize at a later date.

The most frequent sites of metastasis from breast cancer include liver, lung, brain, and bones. Metastatic involvement of the GI tract secondary to a primary breast malignancy is rare. Matsuda et al. reported 73 cases of GI tract spread out of which only 24 involved the colorectal region [2]. Ductal carcinoma of the breast is the most prevalent primary breast cancer subtype followed by invasive lobular cancer. Even though invasive lobular carcinoma accounts for only 5% to 15% of the total breast malignancies, it is the most common histopathological diagnosis in the case of metastasis to the GI tract [3-4]. Upon review of literature, the usual time interval between diagnosis of primary breast cancer to colon metastasis is highly variable ranging from being synchronous to up to 30 years. The mechanism of cancer dissemination is thought to be primarily hematogenous; however, lymphatic and peritoneal spread have also been implicated [8].

Since the spectrum of clinical presentation of metastatic breast cancer to the colon is very wide and maybe non-specific, this poses a significant diagnostic challenge. The manifestations may mimic other acute or chronic inflammatory disorders including inflammatory bowel disease and diverticulitis [9-10]. It is also important to consider that while cancer may masquerade in multiple ways, a second primary GI cancer is much more commonly observed compared to metastatic GI cancer in women with breast cancer [11-12].

Hence, in a patient with a history of breast cancer presenting with symptoms of abdominal discomfort, fatigue, increased abdominal girth, bowel obstruction, palpable abdominal mass, change in bowel habits, microcytic anemia, or at times even subtle vague symptoms should prompt further workup to evaluate for metachronous malignancies. Diagnostic modalities include imaging with CT which may show colonic wall thickening, peritoneal implantation, or nodal involvement. Colonoscopy may reveal suspicious polyps/ masses which should be further evaluated with histopathological analysis and immunohistochemical testing. In the case of metastatic invasive lobular cancer, the immunohistochemical analysis will reveal gross cystic disease fluid protein-15 (GCDFP-15), ER and PR and may even test positive for other antigenic markers including CK 7 and CK20, mucin (MUC) 1, MUC 2, and GCFDP [12].

Another noteworthy characteristic is that this patient had some suspicious enhancement noted on her uterus which confounded the diagnosis in a post-menopausal female making it difficult to discern the exact primary etiology of the malignancy. Also, while CA 125 is routinely elevated in ovarian cancer and CEA is found to be high in colorectal malignancies, both of these may also be elevated in metastatic breast cancer as noted in the case of our patient [13-14].

The prognosis of metastatic breast cancer to the GI tract is dismal with very few patients surviving beyond two years. This case demonstrates the importance of having a high index of suspicion in patients with breast cancer presenting with GI symptoms and/ or symptoms concerning for a malignancy. One of the reasons for the higher mortality may be that the cases are diagnosed at a later stage.

Also of note is that our patient had breast cancer screening but had never had colorectal cancer screening. As per statistics from the National Institute of Health, in 2015, 71.6% women had a screening mammogram, while 63.4% of women got colonoscopy within the screening age group indicating that while women are getting the mammogram, there still exists an 8.2% difference between the colonoscopy and mammogram rate. This means that there is a greater chance that we will be missing out on diagnosing primary colorectal cancer. If this gap was bridged, we would be able to identify a greater number of cases with primary or secondary colorectal cancer at an earlier stage, offer treatment options, and decrease mortality.

## Conclusions

This case describes the importance of considering metastatic breast cancer as a differential in patients with a history of a primary breast malignancy presenting with non-specific abdominal symptoms. Because even though rare, metastatic breast cancer to the GI tract is associated with a much more aggressive disease pattern and dismal outcomes. In our patient, initially, a primary gynecological malignancy was being considered, imaging then suggested a GI malignancy and final biopsy revealed a breast primary showing the diagnostic challenge posed by such cases.
